# Multi-Objective Machine Learning Optimization of Cylindrical TPMS Lattices for Bone Implants

**DOI:** 10.3390/biomimetics10070475

**Published:** 2025-07-18

**Authors:** Mansoureh Rezapourian, Ali Cheloee Darabi, Mohammadreza Khoshbin, Irina Hussainova

**Affiliations:** 1 Department of Mechanical and Industrial Engineering, Tallinn University of Technology, 19086 Tallinn, Estonia; irina.hussainova@taltech.ee; 2 Institut für Materialprüfung, Werkstoffkunde und Festigkeitslehre, Universität Stuttgart, Pfaffenwaldring 32, 70569 Stuttgart, Germany; ch.darabi@gmail.com; 3 Department of Mechanical Engineering, Shahid Rajaee Teacher Training University, Lavizan, Tehran 1678815811, Iran; mkhoshbin@sru.ac.ir

**Keywords:** triply periodic minimal surfaces (TPMS), bone implants, multi-objective optimization, machine learning, artificial neural network (ANN), mechanical property prediction, finite element analysis (FEA), Johnson–Cook failure model, size-specific implant design, Pareto front analysis

## Abstract

This study presents a multi-objective optimization framework for designing cylindrical triply periodic minimal surface (TPMS) lattices tailored for bone implant applications. Using an artificial neural network (ANN) as a surrogate model trained on simulated data, four key properties—ultimate stress (U), energy absorption (EA), surface area-to-volume ratio (SA/VR), and relative density (RD)—were predicted from seven lattice design parameters. To address anatomical variability, a novel implant size-based categorization (small, medium, and large) was introduced, and separate optimization runs were conducted for each group. The optimization was performed via the NSGA-II algorithm to maximize mechanical performance (U and EA) and surface efficiency (SA/VR), while filtering for biologically relevant RD values (20–40%). Separate optimization runs were conducted for small, medium, and large implant size groups. A total of 105 Pareto-optimal designs were identified, with 75 designs retained after RD filtering. SHapley Additive exPlanations (SHAP) analysis revealed the dominant influence of thickness and unit cell size on target properties. Kernel density and boxplot comparisons confirmed distinct performance trends across size groups. The framework effectively balances competing design goals and enables the selection of size-specific lattices. The proposed approach provides a reproducible pathway for optimizing bioarchitectures, with the potential to accelerate the development of lattice-based implants in personalized medicine.

## 1. Introduction

Triply periodic minimal surface (TPMS) lattices are being extensively explored in the field of biomedical engineering, particularly in the design of bone implants and scaffolds [1,2,3]. Their mathematically defined periodic architecture enables the creation of porous structures with high surface area (SA) [4], low density [5], and favorable mechanical properties [6] that closely mimic the complex trabecular and cortical morphology of natural bone [7,8]. Unlike traditional lattice types, TPMS structures, such as Gyroid, Diamond, and Split-P, offer continuous, smooth surfaces that can enhance cell attachment, nutrient transport, and osseointegration [9,10,11,12]. Recent advances in additive manufacturing (AM), particularly selective laser melting (SLM) [13], have made the precise fabrication of such complex TPMS-based scaffolds feasible, enabling customized patient-specific solutions in orthopedic and dental applications [14,15,16].

Although TPMS-based lattices have the potential to be used in bone applications, creating them for biomedical implants requires striking a balance between conflicting goals [17,18]. For instance, maximizing mechanical strength and energy absorption often conflicts with achieving high porosity or optimal surface-to-volume ratios (SA/VR) needed for biological integration [19]. Therefore, multi-objective optimization becomes crucial for identifying lattice designs that balance these properties [19,20]. Classical optimization approaches, while effective, are often computationally expensive or limited in exploring high-dimensional parametric design spaces [21]. Evolutionary algorithms such as the non-dominated sorting genetic algorithm II (NSGA-II) are particularly well-suited to this challenge [17], as they can produce a diverse set of Pareto-optimal solutions representing different optimal compromises [19,22].

A notable limitation in most existing research is the lack of attention to the influence of implant size on design optimization [23]. In clinical practice, implants vary substantially in scale, ranging from small dental screws to large femoral stems [24]. These size variations influence not only mechanical loading conditions but also manufacturability, surface interaction, and biological response [25]. To address this gap, our study introduces a structured classification of TPMS lattice designs into three implant size categories—small, medium, and large—based on their geometric dimensions. Additionally, instead of performing time-consuming simulations, we leverage machine learning (ML) models [26] trained on finite element analysis (FEA) data to efficiently predict key mechanical and geometrical outputs. In particular, artificial neural networks (ANNs) [27] and random forest (RF) regressor are used to enable surrogate modeling, fast optimization, and explainability through SHapley Additive exPlanations (SHAP) analysis [28,29].

While TPMS-based lattices have been widely studied for their mechanical efficiency and manufacturability, existing studies often focus on isolated morphologies, fixed design scales, or purely mechanical targets, offering limited guidance for clinical translation or size-specific implant design. Moreover, few studies have explored the integration of explainable AI methods or multi-objective optimization in large, diverse TPMS datasets. In contrast, this study addresses these gaps by providing a comprehensive design exploration across three TPMS types (Gyroid, Diamond, Split-P) and introducing a clinically meaningful categorization into small, medium, and large implant sizes. This classification facilitates the selection of anatomically relevant designs, supporting patient-specific applications. By coupling ML-based optimization with functionality-focused design and implant size separation, this work contributes new insights into scalable, clinically adaptable implant structures.

The objective of this study is to develop and evaluate an ML-powered, multi-objective optimization framework for designing TPMS-based cylindrical implants tailored to different implant sizes. Our goals are to (1) generate a large-scale dataset of TPMS structures through parametric design and FEA simulation; (2) train and validate ML models capable of accurately predicting key performance metrics such as ultimate stress, energy absorption, SA/VR, and relative density (RD); (3) apply NSGA-II optimization to extract Pareto-optimal lattice designs within medical relevance constraints; (4) analyze the influence of input parameters and implant size on optimization outcomes. By integrating computational design, ML-based modeling, and evolutionary optimization, this study aims to advance the personalized, interpretable, and efficient design of lattice-based bone implants.

## 2. Materials and Methods

### 2.1. Design Automation and Parametric Configuration of TPMS Lattices

In this study, we generated a comprehensive dataset of 3456 cylindrical TPMS lattice structures to evaluate their suitability for bone implant applications. The dataset comprises three surface types—Gyroid, Diamond, and Split-P—selected for their high porosity, manufacturability, and mechanical strength under quasi-static compression test. All structures were modeled as shell-type lattices using mathematical implicit surfaces, and automated geometry generation was performed via a Python interface integrated with nTop (nTop 5.18.2, nTop Inc., New York, NY, USA) [30] software.

**Figure 1:**
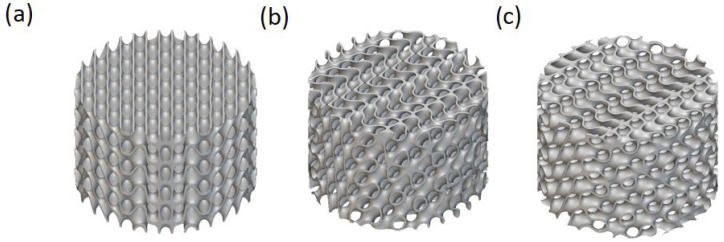
The effect of the unit cell’s rotation angle on the lattice configuration for solid-based Diamond TPMS with a thickness of 0.3, a height of 5 mm, and a diameter of 20 mm with the unit cell sizes of 3, 3, and 4 mm in the X, Y, and Z directions, respectively, for (**a**) 0, (**b**) 30, and (**c**) 60 degrees.

Each design was confined within a cylindrical envelope, and a full-factorial design model was conducted over seven geometric design parameters to investigate their influence on the mechanical response. The number of unit cells along the X-axis (Xcell), Y-axis (Ycell), and Z-axis (Zcell) varied from 3 to 4. The surface shell thickness of the lattice structures was set to 0.2, 0.3, and 0.4 mm. A rotation angle of 0°, 30°, and 60° was introduced to the unit cells about the X-axis. The total height of the cylindrical lattice varied from 8 to 20 mm, and the outer diameter ranged from 5 to 20 mm. Figure 1 shows the unit cell’s effect of the rotation angle of the lattice configuration [31]. Designs violating the geometric stability criterion of height-to-diameter ratio (H/D≥2) were filtered out to prevent buckling, resulting in a final set of 3024 valid structures. For each design, essential geometrical descriptors such as RD, SA, SA/VR, and porosity were calculated using the nTop [30] interface.

### 2.2. Finite Element Analysis (FEA) and Simulation Setup

First, meshing was performed using a uniform surface mesh size of 0.3 mm across all lattice structures, ensuring consistency while balancing accuracy and computational efficiency. Appropriate boundary conditions were then applied: the bottom plate was fully fixed in all degrees of freedom, while the top plate was subjected to a constant downward velocity to simulate uniaxial compression. Contact definitions included hard contact in the normal direction between the lattice and the compression plates, along with tangential penalty frictional contact using a coefficient of 0.1. Additionally, self-contact was enabled to accurately capture strut interactions and post-buckling behavior during the compression simulations. The mechanical performance of each lattice was evaluated under quasi-static compression using the Abaqus/Explicit solver (Abaqus 2022, Dassault Systèmes Simulia Corp., Johnston, RI, USA). The entire simulation pipeline was automated using a Python script that leveraged the Abaqus scripting interface to simplify the process. The shell thickness was assigned by importing the nTop-generated (.odb) mesh surfaces into Abaqus and programmatically setting thickness values based on the corresponding design variables [3,32,33].

The base material used in the simulations was Ti6Al4V alloy, a widely adopted biocompatible material for orthopedic implants [34,35]. The material model incorporated both elastic-plastic deformation and damage evolution to accurately capture its mechanical behavior. The elastic properties were defined with a Young’s modulus of 107.5 GPa, a Poisson’s ratio of 0.3, and a density of 10,750 ${\mathrm{kg}/m}^{3}$. Plastic material properties were derived from tensile stress-strain data reported in [36].

To model failure behavior under compressive loading, the Johnson–Cook failure model was implemented. As the study by Sun et al. [36] did not include failure characteristics in compression, calibrating of the Johnson–Cook damage parameters was performed using the experimental stress–strain response of a Gyroid lattice structure as the validation case study. A set of trial simulations was conducted with varying values of D1=0.005, D2=0.55, and D3=−0.25, while keeping D4 and D5 set to zero. The parameter selected based on the best match to the experimental compression curve was then applied to all lattice simulations to ensure consistency.

### 2.3. Mechanical Property Extraction

Each simulation automatically exported force-displacement data, which was post-processed to compute key mechanical metrics. The ultimate stress (U) was defined as the maximum engineering stress reached during compression. At the same time, energy absorption (EA) was calculated as the area under the stress–strain curve up to a strain of 0.5. Additional metrics, such as Young’s modulus, yield strength, and plateau stress, were also extracted for extended studies, although they were not the primary focus of this work. We selected U and EA as primary objectives to maximize because they directly represent the lattice’s ability to withstand peak loads and absorb mechanical energy [37,38]. Both of which are critical for ensuring structural integrity and shock resistance in load-bearing bone implants [39,40]. The complete dataset of simulated mechanical responses, along with their corresponding geometrical and design parameters, formed the basis for training the machine learning model and conducting multi-objective optimization.

### 2.4. Categorization Based on Implant Size

Recognizing the biomechanical demands of implants for various anatomical locations, the entire dataset was grouped into three categories: small, medium, and large, based on a combination of height and diameter values [41,42,43]. The classification was defined as follows:

- Small: H≤8 and D≤8mm;
- Medium: H≥10 or H≤15 and D≥10 or D≥15mm;
- Large: H>15 or D>15mm.

**Figure 2:**
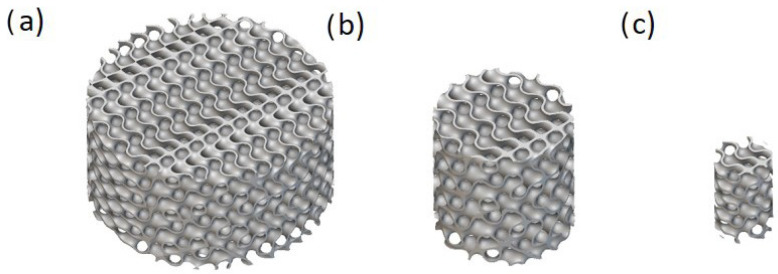
An example of three size categories for solid-based Diamond TPMS with a thickness of 0.3, unit cell sizes of 3, 3, and 4 mm in the X, Y, and Z directions, and the unit cell’s rotation angle of 60 degrees with (**a**) a height of 10 mm and a diameter of 20 mm, (**b**) a height of 10 mm and a diameter of 10, and (**c**) a height of 8 mm and a diameter of 5 mm.

This size-based separation enabled tailored analysis and optimization for various clinical use cases, including dental, cranial, and femoral applications. An example of the size category of the solid-based Diamond lattices is shown in Figure 2.

### 2.5. Feature Pre-Processing

Following the simulation and structural generation stages, a comprehensive set of geometrical features was extracted for the 3024 TPMS lattice from the nTop [30] software. These features included RD, SA, and SA/VR. The RD provides a normalized measure of material usage across varying designs. SA and SA/VR were directly calculated from the shell-type lattice geometry, which is highly relevant in bone implant applications for both mechanical strength and the biological potential for osteointegration [44].

To enable category-specific optimization, labeled lattices—such as small, medium, and large—were introduced based on predefined geometric criteria as mentioned in Section 2.4. This categorization enabled subgroup-specific optimization and facilitated statistical interpretation of the resulting mechanical and geometrical behaviors.

Since the surface type (Gyroid, Diamond, or Split-P) is a categorical feature, it was encoded using one-hot encoding, converting the string-based “Type” feature into three binary variables: $Type_{g}$, $Type_{d}$, and $Type_{s}$, each representing one of the surface types. This encoding facilitated the inclusion of categorical information in regression and ML models without introducing ordinal bias.

All numerical input features—including unit cell counts, shell thickness, rotation angle, height, diameter, and the one-hot encoded surface types—were standardized using a z-score normalization approach. This step was essential to ensure that all features contributed equally to the learning algorithms and to prevent scale dominance from influencing the predictions. The standardization process transformed each input variable to have zero mean and unit variance, allowing the ANN and other machine learning models to converge more efficiently during training.

### 2.6. Predictive Modeling with ANN

We implemented a feedforward ANN to predict four output properties of each lattice: U, EA, SA/VR, and RD. The ANN was constructed using Keras with a TensorFlow backend. It included an input layer with 10–13 features (depending on the encoded types), three hidden layers (128, 64, and 32 neurons, respectively), and a four-neuron output layer with linear activation. The model was trained using the Adam optimizer with a learning rate of 0.001 and mean squared error (MSE) loss.

The dataset of 3024 lattice samples was randomly split into 80% for training (2419 samples) and 20% for testing (605 samples), with all features scaled using the StandardScaler. Model evaluation included $R^{2}$ and mean absolute error (MAE) metrics for each output. The trained ANN model served as a surrogate model in the multi-objective optimization step, enabling rapid prediction of properties for unrecognized designs. K-fold cross-validation was not applied; the model was evaluated on a completely unseen test set, and the consistently high $R^{2}$ values demonstrate its strong generalization performance.

### 2.7. Multi-Objective Optimization

The final step involved optimizing lattice designs for multiple conflicting objectives using NSGA-II. The surrogate ANN model was embedded within an optimization pipeline to accelerate evaluations. Separate optimization runs were executed for each implant size group (small, medium, large) to account for anatomical scaling and constraints. The three objectives were to maximize U, EA, and SA/VR. While RD was not directly optimized, it was predicted and used as a filter. Designs with RD within the ranges of [20–40%] were retained, based on biomedical literature recommending these porosity ranges for bone integration and mechanical compatibility [45,46].

For each group, the optimization search space was defined by the minimum and maximum values of the input variables. The NSGA-II algorithm used a population size of 100, SBX crossover, and polynomial mutation, with a total of 50 generations per group. The resulting Pareto fronts were visualized in both 2D (U vs. EA) and 3D (U, EA, SA/VR) plots, with design diversity and performance clearly illustrated. Following optimization, predicted RD values were added to each Pareto-optimal design using the trained ANN model. The resulting dataset of optimized structures was further analyzed to identify the top-performing lattices for potential fabrication and experimental validation.

### 2.8. Objective Definition

The primary goal of this study was to identify TPMS lattice designs that exhibit optimal mechanical behavior for load-bearing implant applications. Two mechanical properties were selected as objective functions: U and EA. Ultimate stress and energy absorption reflect the lattice’s peak load-carrying capacity and the structure’s ability to dissipate energy during deformation. These metrics are fundamental to implant stability and shock tolerance, particularly in dynamic physiological environments. To explore the relation between strength and toughness, we formulated a two-objective optimization problem where both U and EA were maximized simultaneously. Optimization was performed independently for small, medium, and large implant groups to account for scale-dependent mechanical behavior.

### 2.9. Sensitivity Analysis for Mechanical Outputs

We performed a global sensitivity analysis using the trained ANN model to gain insight into how each design parameter influences the mechanical performance of optimized lattices. We adopted a one-factor-at-a-time (OFAT) approach, where one design variable was varied across its full range while others were held at their mean values. The ANN was then used to predict corresponding changes in U and EA. Sensitivity curves were generated for each input variable (Xcell, Ycell, Zcell, rotation angle, thickness, height, and diameter), and the magnitude of influence was assessed by observing the response gradients. This analysis supports model explainability and provides practical design guidance, highlighting which parameters are most effective to tune when optimizing mechanical performance in TPMS lattices.

### 2.10. Model Explainability with SHAP and RF

To gain insight into the influence of each design parameter on the mechanical and geometrical performance of the TPMS lattices, we employed RF regressors in conjunction with SHAP analysis. While ANN served as the primary surrogate model for multi-objective optimization, RF models were specifically trained on the same dataset to extract insights into the local and global importance of features. SHAP values provide a unified measure of how much each feature contributes to the model’s output, enabling a detailed understanding of complex relationships between input variables and predicted targets. Separate RF models were trained for each output parameter—U EA, SA/VR, and RD—using the scaled design variables as inputs. For each implant size category (small, medium, and large), SHAP summary plots were generated to visualize feature contributions across all samples. This group-wise interpretability enabled us to discern the role of key geometrical features such as wall thickness, unit cell repetition, and lattice orientation in determining performance outcomes. This level of explainability not only validates the surrogate model’s learned behavior but also informs future design exploration in biomedical lattice development.

## 3. Results and Discussion

### 3.1. Distribution and Summary of Lattice Designs

The distribution of lattices among size groups is shown in Table 1. The mechanical and geometrical behavior of TPMS-based lattices was analyzed across three implant size categories—small, medium, and large—using both boxplots and kernel density estimation (KDE) plots. This analysis includes RD, SA/VR, U, and EA.

**Figure 3:**
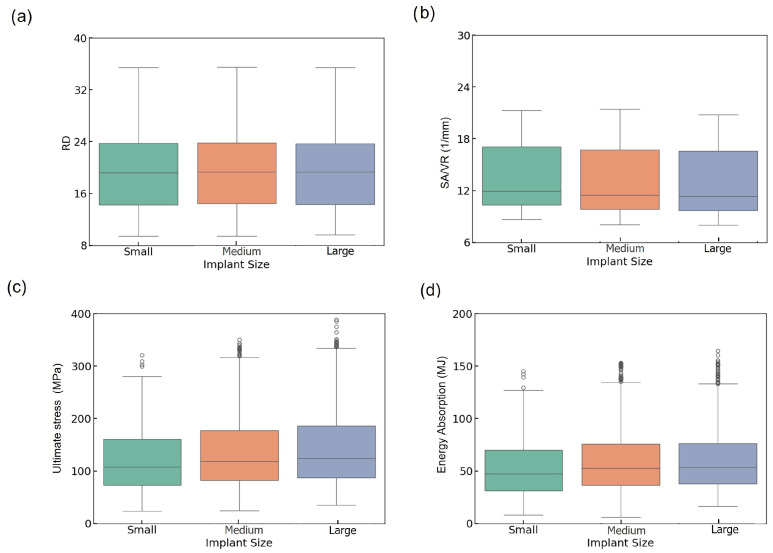
Boxplots of (**a**) RD, (**b**) SA/VR, (**c**) U, and (**d**) EA across small, medium, and large implant sizes.

Figure 3a presents the boxplots of RD across the three implant size groups. The distributions appear consistent, with all groups showing similar ranges and a median around 19–20% and maximum values (~35%), with minor variations in the lower bounds. This suggests that implant size has a limited impact on RD variation, confirming that the design parameter sweep effectively maintains control over RD values across all categories. The uniformity in RD across implant sizes suggests that RD was effectively controlled during lattice design, ensuring the proper comparison of mechanical performance across groups. This strengthens the validity of implant size-based comparisons without density-induced bias.

The boxplot of SA/VR as a critical factor in predicting implant integration with biological tissue [47] follows a comparable trend (Figure 3b), indicating slightly higher values and a greater spread in the small group compared to the medium and large groups. Despite similar medians, the small group exhibits a wider upper whisker and a higher number of outliers, indicating enhanced geometrical diversity. This increased SA/VR variability in smaller implants may enhance surface functionality, which is particularly desirable for osseointegration performance.

From a biological perspective, RD and SA/VR are critical geometric factors that influence the interaction between scaffolds and tissues [48,49]. RD governs porosity, which directly affects cell migration, neovascularization, and nutrient flow, thereby facilitating bone ingrowth [46]. Studies have suggested that a porosity corresponding to an RD of 10–40% aligns well with the native trabecular bone environment [45]. Likewise, SA/VR is a measure of the surface landscape available for cell attachment and osteointegration. Higher SA/VR ratios have been linked to enhanced cell adhesion, proliferation, and osteogenic activity, making it a relevant target for optimizing scaffold performance [46].

In Figure 3c, the boxplot of U shows a progressive increase from small to large implants. Median U values are higher in medium and large implants than in the small group, and the range of stress values broadens with increasing size. The large group achieves a higher upper range (~370 MPa), and a greater number of high-value outliers, indicating higher mechanical strength. The ascending trend in U from small to large implants underscores the role of physical volume and structure reinforcement in bearing higher loads. This suggests that larger implants tend to be more robust mechanically, although their increased variability may result from more complex failure mechanisms, such as local buckling.

The boxplot of EA is represented in Figure 3d, where medium and large implants exhibit higher median values and broader distributions compared to small ones. The median EA increases from small (~47 MJ) to large (~55 MJ), exhibiting more high-energy outliers, which suggests improved shock resistance in larger lattice structures.

**Figure 4:**
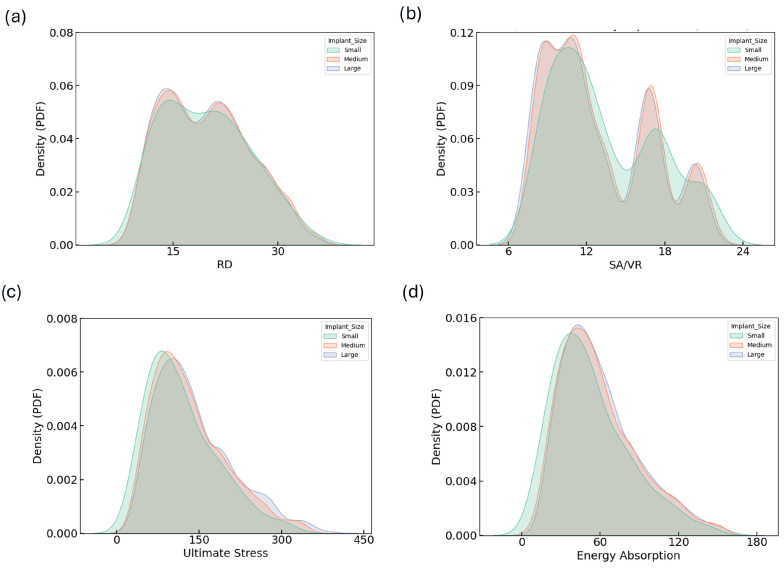
KDE plots showing the distribution of (**a**) RD, (**b**) SA/VR, (**c**) U, and (**d**) EA across small, medium, and large implant size categories. These plots illustrate how geometric and mechanical properties vary by implant size group. While the general distribution trends are similar, subtle shifts in peak positions and tails indicate that implant size influences the achievable design space and mechanical performance envelope.

KDE plots provide a detailed view of the distributions of geometric and mechanical properties across implant size groups. Figure 4 illustrates the KDE plots for RD, SA/VR, U, and EA across all implant sizes. The RD distributions (Figure 4a) show notable overlap among small, medium, and large categories, all peaking consistently around 18–20%, indicating well-controlled design uniformity regardless of implant size. In contrast, the SA/VR plot (Figure 4b) reveals a broader and more varied distribution in small implants, characterized by multiple peaks, whereas medium and large implants exhibit narrower, centralized curves. This highlights the potential of small implants to provide enhanced surface complexity, beneficial for biological integration.

The distribution of ultimate stress exhibits a unimodal pattern skewed to the right for all groups, with peaks between 100 and 150 MPa. The wider tail in the large group reflects a broader range of high-strength designs. Similarly, energy absorption distributions peak around 50–60 MJ for all sizes, but the large group again displays a more dispersed curve, suggesting greater design versatility. Figure 4d shows the KDE plot for EA. These consistent trends in SA/VR, U, and EA justify the need for independent optimization across implant sizes and support the strategic exploration of size-dependent design benefits.

### 3.2. Predictive Modeling with ML

**Figure 5:**
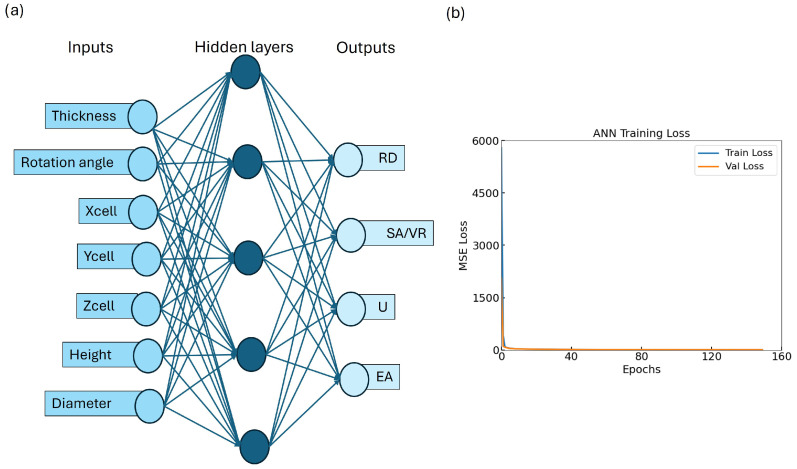
Overview of model performance: (**a**) ANN architecture; (**b**) training–validation loss curve showing convergence and minimal overfitting.

To assess the effectiveness of the trained ANN for predicting the mechanical and geometrical properties of TPMS lattices, we evaluated its performance on both the training and validation datasets. The ANN architecture, depicted in Figure 5a, maps seven design variables to four target outputs (RD, SA/VR, U, EA) via a fully connected multi-layer structure. The training and validation MSE loss curves are shown in Figure 5b. A rapid convergence is observed within the first 20 epochs, followed by stable low error across 150 epochs. The close alignment between training and validation loss indicates minimal overfitting, suggesting that the ANN generalizes well to unseen data.

**Figure 6:**
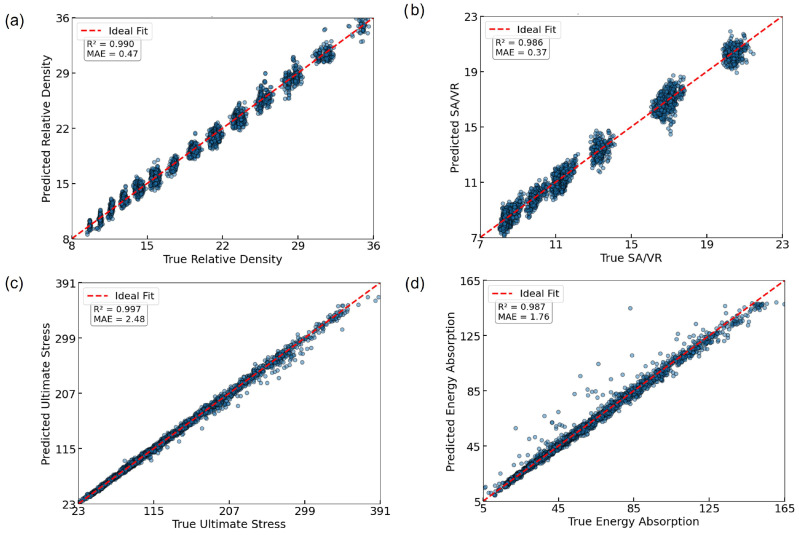
Regression plots showing true vs. predicted values with $R^{2}$ and MAE metrics for all four outputs: (**a**) RD; (**b**) SA/VR; (**c**) U; (**d**) EA.

The predictive performance of the model was further validated using regression plots comparing actual versus predicted values for each output, as shown in Figure 6. The ANN achieved excellent agreement across all targets, with $R^{2}$ values of 0.997 for U, 0.987 for EA, 0.986 for SA/VR, and 0.990 for RD. MAE values remained low in all cases (≤2.5), confirming the model’s ability to approximate highly nonlinear relationships between input parameters and resulting properties with high fidelity.

It is important to note that the trained ANN model is specific to the three TPMS morphologies used in this study (Gyroid, Diamond, and Split-P) and is not directly generalizable to other TPMS types. However, the framework is extensible and can accommodate additional geometries by incorporating their simulation data and retraining the model accordingly.

**Figure 7:**
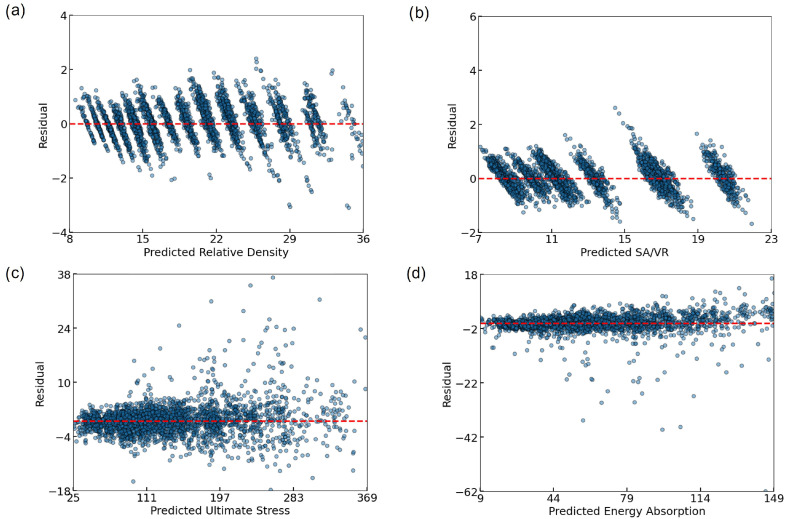
Residual plots illustrating the prediction errors of the ANN model for (**a**) RD, (**b**) SA/VR, (**c**) U, and (**d**) EA. Each subplot shows the difference between the predicted and actual values across the test set, with a dashed red line indicating zero residual. The generally tight clustering around the zero line suggests minimal bias and strong model generalization, although some dispersion is observed at higher output values, particularly in mechanical properties.

To further evaluate the prediction behavior of the ANN, residual plots were generated for each target variable (Figure 7). All residuals were tightly clustered around zero, indicating low bias and good predictive alignment. For RD (Figure 7a) and SA/VR (Figure 7b), a discrete banding pattern emerged, due to the limited granularity of the input parameters. Despite this, the residuals remained within a narrow range. Ultimate stress (Figure 7c) exhibited a mild heteroscedastic trend, with increasing variance at higher stress values, yet without significant systematic error. The energy absorption plot (Figure 7d) revealed slightly heavier-tailed errors, particularly underestimation at higher values, but the central distribution remained balanced. Overall, the residuals validate the ANN model’s robustness across all four outputs.

Furthermore, the reliability of the simulation data used for training the machine learning model was confirmed in our previous work [31]. Three Gyroid TPMS designs—selected initially from the same dataset of 1008 Gyroid structures used in this study—were fabricated using SLM and tested under quasi-static compression. The experimental results closely matched the simulation outcomes, validating the accuracy of the simulation pipeline and the material model employed here.

Although SHAP analysis was not conducted separately for each implant group, post-optimization SHAP analysis (discussed in Section 2.10) provided valuable insight into the influence of features in the high-performing Pareto-optimal designs. Given the strong predictive performance of the ANN model, it was considered suitable as a surrogate model for downstream optimization tasks. The integration of machine learning not only accelerated the prediction process but also allowed for the exploration of a vast design space without the computational cost of repeated FEA simulations.

### 3.3. Multi-Objective Optimization Outcomes

To efficiently explore the complex design space of TPMS-based lattices and identify optimal geometries for biomedical implants, an optimization framework was developed. A previously trained ANN is used as a surrogate model, enabling rapid prediction of mechanical and geometrical properties for any given combination of design variables. This approach significantly reduced the computational cost compared to running finite element simulations for each candidate design. The multi-objective optimization was conducted using NSGA-II, which is well-suited for solving problems involving multiple conflicting objectives. The optimization simultaneously aimed to maximize three key objectives of U, EA, and SA/VR. These targets aim to achieve the dual goals of enhancing mechanical performance and promoting osseointegration. The optimization results in a total of 105 lattice designs.

Following the prediction of objective values by the ANN, a post-optimization filtering step was applied to ensure biomedical applicability. Specifically, only designs with predicted RD within the ranges of [20–40%] were retained. These thresholds are informed by empirical studies that link RD to bone ingrowth and mechanical compatibility with native tissue. The entire optimization process was stratified by implant size. Separate NSGA-II runs were executed independently for the small, medium, and large implant size categories. This grouping enabled the identification of size-specific Pareto-optimal solutions, taking into account anatomical differences in the application context.

### 3.4. Pareto Front Visualizations and Trade-Off Analysis

**Figure 8:**
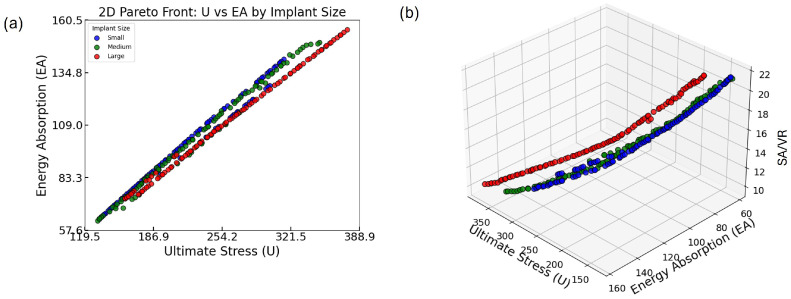
(**a**) Two-dimensional Pareto front showing the trade-off between U and EA across all implant sizes. (**b**) Three-dimensional Pareto front incorporating SA/VR, illustrating the multi-objective optimization landscape and size-specific distribution of optimal lattice designs.

To evaluate the effectiveness of the multi-objective optimization and explore the trade-offs among mechanical and geometrical objectives, Pareto-optimal solutions were visualized in both 2D and 3D spaces, as presented in Figure 8. These visualizations enable a comprehensive comparison across implant size categories—small, medium, and large—and provide critical insights into feasible design compromises that are highly relevant to practical applications, particularly in the context of bone implants.

Figure 8a presents the 2D Pareto fronts for the three implant size groups based on two key mechanical objectives: U and EA. Each point represents a Pareto-optimal lattice design, meaning that no other design in the solution set improves one objective without compromising the other. A clear, strong positive correlation is observed between U and EA across all groups: as ultimate stress increases, so does energy absorption. This trend highlights a favorable synergistic relationship between load-bearing capacity and energy dissipation, both of which are crucial for orthopedic implants that must withstand mechanical loading while minimizing damage transfer to the surrounding bone.

Notably, the Pareto front for the small group extends slightly further in the upper-right corner of the plot, indicating that some of the highest-performing designs in terms of both U and EA belong to this group. This suggests that within the explored design space, small implants are capable of achieving superior combinations of strength and toughness. However, the medium and large groups offer broader distributions along the trade-off curve, which may be beneficial in scenarios where moderate performance in both metrics is acceptable but geometrical or anatomical constraints require larger implant volumes. Such design flexibility is essential for addressing diverse patient-specific requirements.

The analysis is further extended in Figure 8b by incorporating SA/VR as a third optimization objective. This 3D Pareto plot provides a multidimensional view of design performance, revealing how mechanical objectives (U and EA) interact with surface-based geometrical characteristics (SA/VR).

The plotted points show that while U and EA maintain a consistent positive correlation, SA/VR does not always follow the same trend. In fact, designs that maximize U and EA tend to cluster around mid-range SA/VR values. Conversely, those with the highest SA/VR often show a compromise in at least one mechanical property. This indicates that although it is possible to achieve strong mechanical performance and adequate SA/VR simultaneously, pushing one objective to its extreme can detract from the others. In practical terms, this reveals that optimal designs for osseointegration may require a deliberate trade-off, balancing surface efficiency with mechanical integrity.

Additionally, the 3D distribution of points shows clear stratification based on implant size. Each implant group occupies a distinct region in the optimization space, implying that the feasible design landscape and performance frontiers differ with implant volume. This finding reinforces the importance of conducting size-specific optimization. By tailoring the optimization process to each implant category, it is possible to select the most suitable set of designs for specific anatomical applications, thereby enhancing clinical outcomes.

Together, the 2D and 3D Pareto fronts serve as a visual and analytical tool for understanding the complex interplay between strength (U), toughness (EA), and osseointegration potential (SA/VR). The frontiers confirm that simultaneously maximizing all three objectives is infeasible, but they offer a well-distributed spectrum of non-dominated solutions from which designers can select the most clinically suitable options.

This comprehensive optimization and visualization approach validates the effectiveness of the multi-objective framework employed in this study. It also emphasizes the necessity of stratified design strategies when developing TPMS-based bone implants. By carefully analyzing these relations, decision-makers—whether engineers or clinicians—can align design priorities with clinical constraints, advancing the development of highly functional, personalized bone implant solutions.

### 3.5. RD Filtering and Post-Optimization Predictions

To ensure biomedical relevance—particularly for bone tissue engineering applications where the interplay between mechanical stability and porosity is critical—a post-optimization filtering step was applied based on predicted RD. Designs with RD values falling within the range of 20–40% were retained, corresponding to the acceptable spectrum for mimicking trabecular bone structures. This range captures the lower threshold required for mechanical performance and the upper bound of porosity beneficial for osseointegration.

**Figure 9:**
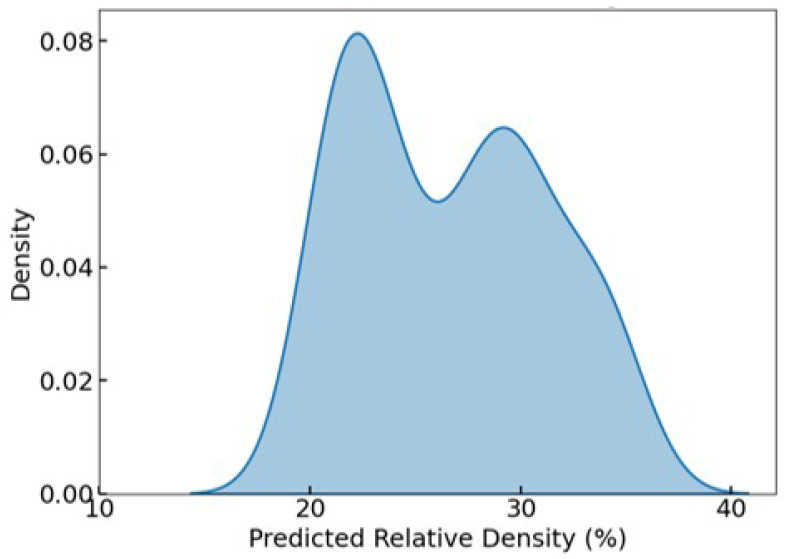
KDE of predicted RD after filtering, showing a bimodal distribution with all values constrained between 20 and 40%, aligning with the targeted range for trabecular bone analogs.

Figure 9 illustrates the KDE of the predicted RD distribution after filtering. The plot reveals a bimodal distribution, with two pronounced peaks centered at approximately 22% and 30%, indicating that the optimization process yielded a diverse yet targeted set of solutions. This bimodal pattern suggests that the surrogate-assisted optimization did not converge to a single narrow band of designs, but instead explored multiple favorable design regions within the target RD window. The absence of values below 20% and above 40% further confirms the effectiveness of the filtering criteria.

This RD-focused filtering process reinforces the practical viability of the retained Pareto-optimal lattices, ensuring that all final candidates fall within a biomechanically and biologically acceptable density range for bone implant applications.

### 3.6. Sensitivity Analysis Results

**Figure 10:**
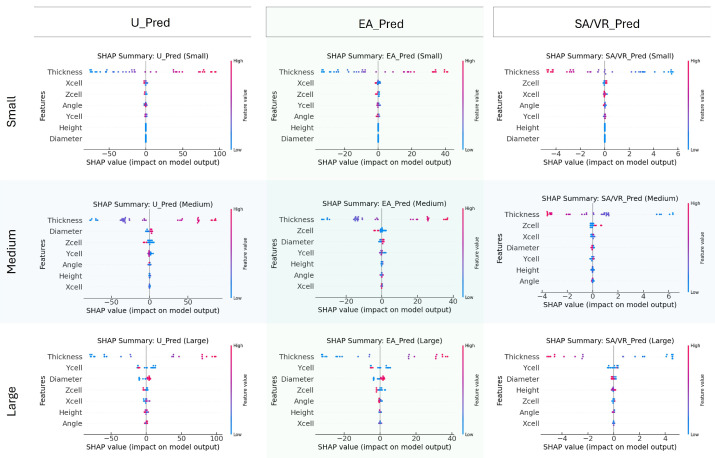
SHAP summary plots for predicting U, EA, and SA/VR across small, medium, and large implant groups. Each plot shows the influence of seven design features on the ANN output, with color indicating feature value (blue = low, red = high) and x-axis position reflecting the impact. Results highlight how feature importance varies with implant size.

To interpret the contribution of each design parameter to model predictions, SHAP values were computed for three target outputs, U, EA, and SA/VR, stratified by implant size. Figure 10 visualizes these results, allowing a comparative interpretation of feature importance and effect direction. For ultimate stress, thickness is consistently the most influential feature across all implant sizes, exerting a strong positive influence when its value is high. In medium and large implants, diameter and Zcell also play prominent roles, indicating that both wall dimension and unit cell complexity contribute significantly to stress-bearing capacity. Interestingly, in the small group, spatial dimensions such as Xcell and Zcell hold more importance compared to Diameter, suggesting that geometric layout dominates strength behavior at smaller scales.

Regarding EA, thickness again emerges as the dominant variable across all size groups, reinforcing its dual contribution to both stiffness and energy dissipation. The influence of Ycell and Zcell increases in the Medium and Large categories, implying that energy dissipation capacity benefits more from structural span and complexity in larger implants. The spread of SHAP values for these features shows a broad and nonlinear impact pattern.

For SA/VR, the most influential feature remains Thickness, but with a consistently negative SHAP trend, affirming that thinner structures yield higher SA/VR. This inverse relationship is crucial for osseointegration-focused designs. Additionally, Zcell and Ycell demonstrate notable importance, particularly in Medium and large groups, highlighting their contribution to surface complexity.

In summary, the SHAP analysis emphasizes that thickness is a universally dominant parameter across all targets and implant sizes. However, the relative impact of other geometric variables, such as diameter, Zcell, and Ycell, varies by implant size, indicating scale-dependent sensitivity. These insights support the need for size-specific design strategies and provide interpretability for surrogate model behavior within the optimization workflow.

## 4. Conclusions

This study presents a comprehensive framework that combines the ANN and NSGA-II algorithms to optimize cylindrical TPMS lattice structures for bone implant applications. The primary objectives were to simultaneously maximize mechanical performance—ultimate stress and energy absorption—and geometric efficiency, as characterized by the SA/VR, while maintaining a biologically relevant range of RD between 20% and 40%.

A robust surrogate model was developed using an ANN trained on 1008 TPMS designs, capturing complex nonlinear relationships between seven design parameters and four target outputs with excellent predictive accuracy ($R^{2}$ > 0.98 for all targets). Model interpretation using SHAP analysis revealed that Thickness was the most influential parameter across all outputs. At the same time, features such as Zcell, diameter, and Ycell displayed varying importance depending on implant size and target property.

The multi-objective optimization process identified 105 Pareto-optimal lattice designs across small, medium, and large implant categories, which were subsequently filtered down to 75 based on the RD constraint. Pareto front visualizations revealed a strong positive correlation between U and EA, while highlighting trade-offs with SA/VR. Notably, the small implant group offered designs with superior mechanical synergy, whereas large implants provided broader design diversity, especially in SA/VR and EA.

Statistical analyses, including boxplots and KDE plots, demonstrated consistent trends across implant sizes and reinforced the scale-dependent nature of performance distributions. Residual diagnostics further confirmed the ANN’s generalizability and low prediction bias, validating its reliability for guiding optimization.

Overall, the findings underscore the effectiveness of combining ML with evolutionary optimization for accelerating the design of high-performance, size-specific TPMS bone implants. The proposed framework not only enables efficient exploration of large design spaces but also provides interpretable insights for balancing competing objectives in biomedical lattice design. The ML model presented in this study is trained on simulation outputs derived from the properties of Ti6Al4V material; the predictions and optimizations are therefore material-specific. However, the framework itself is transferable. By resimulating the lattice dataset using alternative material models and retraining the predictive model, the same methodology can be applied to other clinically relevant materials.

In future work, we aim to fabricate and experimentally test a selected set of Pareto-optimal TPMS lattices across different implant size categories. Using SLM with Ti6Al4V powder, we will manufacture small- to large-sized implant prototypes and perform quasi-static compression testing to further validate the simulation-based predictions. Although RD and SA/VR were used as structural proxies for biological behavior, future work will incorporate biological performance indicators, utilizing Computational Fluid Dynamics (CFD) and experimental cell studies, to better capture the osseointegration potential in lattice design.

Despite these contributions, this study has some limitations. The models were trained on simulated data using Ti6Al4V and did not account for uncertainty in material properties or boundary conditions. Additionally, biological processes such as osseointegration or fluid transport were not directly modeled. These aspects are part of our ongoing and future work, including experimental validation, biological simulation integration, and patient-specific design based on CT data.

## Figures and Tables

**Table 1:** Distribution of lattice designs by TPMS type and implant size.

Lattice Type	Small	Medium	Large	Total
Gyroid	72	504	432	1008
Diamond	72	504	432	1008
Split-P	72	504	432	1008
Total	216	1512	1295	3024

## Data Availability

Some or all of the data and models that support the findings of this study are available from the corresponding author upon request.
